# Cost analysis of insulin degludec in comparison with insulin detemir in treatment of children and adolescents with type 1 diabetes in the UK

**DOI:** 10.1136/bmjdrc-2019-000664

**Published:** 2019-09-03

**Authors:** Nandu Thalange, Jens Gundgaard, Witesh Parekh, Deniz Tutkunkardas

**Affiliations:** 1 Al Jalila Children’s Hospital, Dubai, United Arab Emirates; 2 Novo Nordisk A/S, Søborg, Denmark; 3 European HEOR, Novo Nordisk Ltd, Gatwick, UK

**Keywords:** degludec, detemir, cost analysis, ketosis, basal insulin

## Abstract

**Objective:**

With healthcare systems under increasing financial pressure from costs associated with diabetes care, it is important to assess which treatments provide clinical benefits and represent best value. This study evaluated the annual costs of insulin degludec (degludec) versus insulin detemir (IDet) in children and adolescents with type 1 diabetes (T1D) in the UK.

**Research design and methods:**

Using data from a randomized, treat-to-target, non-inferiority trial—BEGIN YOUNG 1—annual costs with degludec versus IDet in children and adolescents aged 1–17 years with T1D were estimated, as costs of these insulins and hyperglycemia with ketosis events. Analyses by age group (1–5, 6–11 and 12–17 years) and scenario (no ketosis benefit, no dose benefit, hyperglycemia with ketones >0.6 and >3.0 mmol/L and the additional costs of twice-daily IDet in 64% of patients) were also performed.

**Results:**

The mean annual cost per patient was estimated as £235.16 for degludec vs £382.91 for IDet, resulting in an annual saving of £147.75 per patient. These substantial cost savings were driven by relative reductions in the frequency of hyperglycemia with ketosis and basal insulin dose with degludec versus IDet. Annual savings in favor of degludec were observed across each age group (£122.63, £140.59 and £172.50 for 1–5, 6–11 and 12–17 years age groups, respectively). Five scenario analyses further demonstrated the robustness of the results, which included no ketosis or dose benefits in favor of degludec.

**Conclusions:**

Degludec provides appreciable annual cost savings compared with IDet in children and adolescents with T1D in a UK setting. While a cost-effectiveness analysis could incorporate the health impact of treatment complications better than the present cost analysis, the strong generalizability of the data from this study suggests that degludec can help healthcare providers to maximize health outcomes despite increasingly stringent budgets.

**Key messages**

**What is already known about this subject?**

- The BEGIN YOUNG 1 trial demonstrated that insulin degludec (degludec) is associated with significantly lower rates of hyperglycemia with ketosis and a 30% lower basal insulin dose compared with insulin detemir (IDet), and degludec has previously been identified as cost-effective compared with insulin glargine 100 and 300 units/mL in adults with type 1 or type 2 diabetes in the UK.

**What are the new findings?**

- The mean annual cost per patient with type 1 diabetes was estimated as £235.16 for those receiving degludec vs £382.91 for those receiving IDet, resulting in an annual saving of £147.75 per patient.
- This annual saving in favor of degludec was observed across three separate age groups (£122.63, £140.59 and £172.50 for 1–5, 6–11 and 12–17 years age groups, respectively), representing early childhood through to late adolescence.
- Five scenario analyses further demonstrated the robustness of these results, which included the adjustment of parameters so that no ketosis or dose benefits in favor of degludec were considered.

**How might these results change the focus of research or clinical practice?**

- The present analysis shows that degludec also provides appreciable annual cost savings (£147.75 per patient) compared with IDet in children and adolescents with type 1 diabetes in a UK setting, which are driven by the relative reductions in the frequency of hyperglycemia with ketosis and basal insulin dose.
- The cost savings achieved, along with the clinical benefits of degludec versus IDet, will evidently facilitate healthcare providers’ ability to maximize health outcomes with restricted budgets.

## Introduction

Healthcare systems are under increasing financial pressure from diabetes care costs.^1^ The global prevalence of diabetes is increasing and consequently expenditure on diabetes is projected to rise substantially in the next two decades.^1^ In the UK, the cost associated with diabetes was approximately £23.7 billion in 2010/2011, and is estimated to rise to £39.8 billion by 2035/2036.^2^

Type 1 diabetes (T1D) is characterized by the autoimmune destruction of pancreatic beta-cells, causing insulin deficiency that prevents the absorption and utilization of glucose,^3^ and typically presents in children and adolescents.^4^ In this patient population, insulin therapy is required immediately following diagnosis.^4^ An appreciable burden on healthcare services arises from acute complications of diabetes—namely hypoglycemia, hyperglycemia and diabetic ketoacidosis (DKA).^5–7^ Hypoglycemia results from relative insulin excess and severe hypoglycemia, which can lead to coma, seizures and even death, requires urgent intervention to prevent serious harm and results in increased healthcare resource use—both immediately, and in the aftermath.^6^ Conversely, relative insulin deficiency leads to hyperglycemia and lipolysis; if unchecked, it may progress to the formation of blood ketones (ketosis) and ultimately, DKA.^7^ Both ketosis events and DKA are associated with high healthcare resource costs arising from doctor visits, ambulance use and hospitalization, including the need for intensive care.^5 7^ Accordingly, a key goal of insulin therapy is to maintain optimal blood glucose levels and prevent the development of these acute diabetes-related complications.^8 9^ New-generation long-acting insulin analogs with improved pharmacokinetic and pharmacodynamic profiles have been developed, and these have the potential to reduce both hypoglycemia and ketosis.^10^

One such new-generation long-acting insulin analog, insulin degludec (degludec), is a basal insulin with a duration of action exceeding 42 hours at steady state and a flat and stable glucose-lowering effect.^11–13^ The clinical benefits of degludec versus insulin detemir (IDet) in children and adolescents (1–17 years of age) with T1D were investigated in BEGIN YOUNG 1. This 26-week, phase IIIb, open-label, multinational, parallel-group, randomized, treat-to-target, non-inferiority trial (with a 26-week extension) compared the efficacy and safety of degludec once daily with that of IDet once daily or twice daily, both in combination with mealtime insulin aspart (IAsp).^14^ In this patient population, at equivalent glycemic control, degludec was associated with a similar rate of hypoglycemia and significantly lower rates of hyperglycemia with ketosis and a 30% lower basal insulin dose, compared with IDet.^14^

With increasing requirements from payers and decision makers to use resources wisely, it is important to assess which treatments provide clinical benefits and consider the best value for the resources used. Therefore, a robust economic evaluation is required based on clinical data. The aim of the present study was to evaluate the annual costs of degludec versus IDet as basal insulin therapy in children and adolescents with T1D, from the perspective of the UK National Health Service (NHS), using data from the BEGIN YOUNG 1 trial.

## Methods

### Main cost analysis

Mean annual costs associated with basal insulins and hyperglycemia with ketosis events for the treatment of degludec or IDet in children and adolescents aged 1–17 years with T1D were calculated. Costs associated with hypoglycemia were not included in the present study as the rates of hypoglycemia had been found not to differ significantly between degludec and IDet.^14^ Annual costs of the basal insulins were calculated as unit costs multiplied by number of units per day multiplied by 365.25, and annual costs of events of hyperglycemia with ketosis were calculated as cost of a single event multiplied by number of these events per patient-year of exposure (PYE). All calculations were performed using Microsoft Excel 2016.

### Clinical parameters

The basal insulin dose ratio and rate ratio (RR) of hyperglycemic events (plasma glucose (PG) >14 mmol/L (252 mg/dL)) with elevated ketones>1.5 mmol/L (27.0 mg/dL) experienced by patients treated with degludec versus IDet during the whole 52-week period of the BEGIN YOUNG 1 study were used in this cost analysis (table 1A).^14^ The dose (units/day) of IDet was calculated as 0.55 units/kg (dose of IDet at end of the BEGIN YOUNG 1 trial)^14^ multiplied by the average weight (37.9 kg) of all patients (table 1A). The dose (units/day) of degludec was estimated by multiplying the daily dose ratio of 0.7 from BEGIN YOUNG 1 by the calculated daily dose of IDet (table 1A).^14^ The number of hyperglycemic events with ketones >1.5 mmol/L (27.0 mg/dL) per PYE for degludec was estimated by multiplying the RR of 0.41 from BEGIN YOUNG 1 by the number per PYE for IDet (table 1A).^14^

**Table 1:** Clinical parameters used in the (A) main cost analyses and (B) sensitivity analyses

Clinical inputs	IDet	Ratio (degludec/IDet)	Degludec (estimated)
**(A)**			
Basal insulin dose (units/day)			
All patients (n=273)	20.85	0.70	14.60
Rate of hyperglycemia with ketosis (number of events per PYE)			
Ketones >1.5 mmol/L (27.0 mg/dL)	1.10	0.41	0.45

Clinical inputs	IDet	Ratio (degludec/IDet)	Degludec (estimated)
**(B)**			
Basal insulin dose (units/day)			
1–5 years of age (n=63)	9.85	0.70	6.90
6–11 years of age (n=112)	17.71	0.70	12.40
12–17 years of age (n=98)	31.68	0.70	22.18
Rate of hyperglycemia with ketosis (number of events per PYE)			
Ketones >0.6 mmol/L (10.8 mg/dL)	6.14	0.44	2.70
Ketones >3.0 mmol/L (54 mg/dL)	0.23	0.28	0.06

N=number of patients completing main trial and extension period (total 52 weeks) from the BEGIN YOUNG 1 study.^14^ All treatment differences associated with the dose ratios and rate ratios were significant. The basal insulin dose ratio, number of hyperglycemic events with ketones >1.5 mmol/L (27.0 mg/dL) per PYE with IDet and the corresponding rate ratio with degludec vs IDet were taken from BEGIN YOUNG 1.^14^ The numbers of hyperglycemic events with ketones >0.6 mmol/L (10.8 mg/dL) and >3.0 mmol/L (54 mg/dL) per PYE, and the corresponding rate ratios with degludec vs IDet were retrieved from a secondary analysis of two phase IIIb trials investigating degludec and degludec with insulin aspart vs IDet.^16^ The number of hyperglycemic events with ketosis per PYE for degludec were estimated as the number per PYE for IDet multiplied by the corresponding rate ratios. The dose (units/day) of IDet was calculated as 0.55 units/kg (dose of IDet at the end of the BEGIN YOUNG 1 trial)^14^ multiplied by the average weight (37.9 kg) of all patients (Table 1A). The dose (units/day) of degludec was estimated by multiplying the daily dose ratio of 0.7 from BEGIN YOUNG 1^14^ by the calculated daily dose of IDet.

IDet, insulin detemir; PYE, patient-year of exposure.

### Cost data

The cost of insulin was calculated based on prices published in Monthly Index of Medical Specialities, June 2018.^15^ Degludec (Tresiba 100 units/mL in FlexTouch pen) was £46.60 for 1500 units, resulting in a cost/unit of £0.031. IDet (Levemir 100 units/mL in Penfill pen) was £42.00 for 1500 units, resulting in a cost/unit of £0.028.

**Figure 1:**
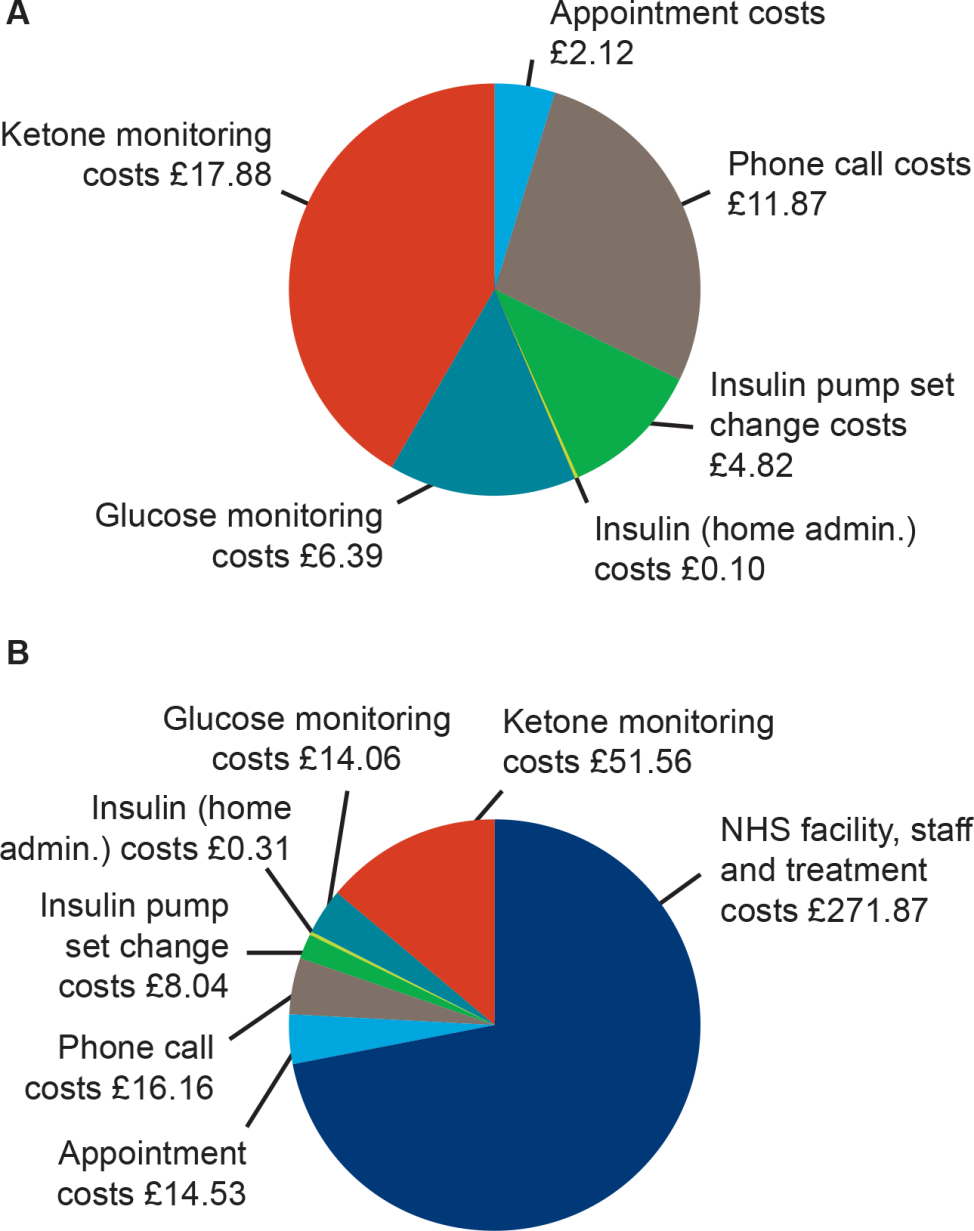
Direct costs associated with hyperglycemia with ketosis events in children and adolescents treated at (A) home only £43.19 and (B) home and NHS facilities £373.53. NHS, National Health Service.

The direct costs associated with a single hyperglycemia with ketosis event treated at home only, or both at home and in NHS facilities, and the weighted average of the direct costs of all hyperglycemia with ketosis events have been previously calculated, based on resource costs estimated from UK data sources and results from a quantitative survey in adult patients, pediatric carers and healthcare professionals.^7^ For the present study, these resource costs were updated with the values current in 2018 (online supplementary table 1). By using the same methodology as the previous calculation^7^ and the updated resource costs, the direct costs associated with treating a single hyperglycemia with ketosis event in children and adolescents treated at home only, or both at home and in NHS facilities were estimated as £43.19 and £376.53, respectively (figure 1). Based on the survey results, the weighted average of all direct costs of a hyperglycemia with ketosis event in children and adolescents was estimated as £154.30, assuming 33% of pediatric hyperglycemia with ketosis events required some level of treatment in NHS facilities, with 67% requiring only home treatment.^7^

10.1136/bmjdrc-2019-000664.supp1 Supplementary data

### Sensitivity analyses

#### Age group analyses

Participants in BEGIN YOUNG 1 were stratified into three age groups: 1–5, 6–11 and 12–17 years, to calculate the mean annual costs of treatment with degludec or IDet together with hyperglycemia with ketosis events experienced. The dose (units/day) of IDet was calculated as 0.55 units/kg (end of trial dose of IDet)^14^ multiplied by the average weight of patients in each age group (17.9, 32.2 and 57.6 kg for the 1–5, 6–11 and 12–17 years age groups, respectively; table 1B). The overall dose ratio (of 0.7) of degludec versus IDet was applied to perform similar calculations to estimate the dose (units/day) of degludec for the three age groups. The rest of the clinical parameters and cost data were the same as those used for the main analysis (table 1B).

#### Scenario analyses

Five varying scenarios were hypothesized, to serve as sensitivity analyses with which to estimate the annual costs of degludec versus IDet in the overall cohort of children and adolescents with T1D, using available data and plausible assumptions.

In the scenarios of no ketosis and no dose benefits, a RR and dose ratio of 1 was assumed, to calculate the number of ketosis events and the daily dose of degludec, respectively.

In scenarios of ketosis with ketone concentrations of >0.6 mmol/L (10.8 mg/dL) or >3.0 mmol/L (54.0 mg/dL) as cut-offs, the number of hyperglycemia with ketosis events per PYE for IDet and the corresponding RRs with degludec versus IDet (table 1B) were taken from a secondary analysis of two phase IIIb trials investigating degludec and degludec with IAsp versus IDet.^16^ These data were used to calculate estimates of the corresponding number for degludec.

The cost of a single hyperglycemic event with ketones >0.6 mmol/L (10.8 mg/dL) was calculated as £43.19, assuming that these events were all treated at home only (figure 1). The cost of a single hyperglycemic event with ketones >3.0 mmol/L (54.0 mg/ dL) was calculated as £376.53, assuming that these events required treatment both at home and in NHS facilities (figure 1).^7^ In the last scenario, the costs of additional healthcare resources required (needles and self-measured blood glucose (SMBG) tests) with 64% of patients administering IDet twice daily, observed in BEGIN YOUNG 1,^14^ were also taken into account.^14^ The costs of needles and SMBG tests were based on the Monthly Index of Medical Specialities June 2018.^15^ Needle choice was assumed to be the same for both basal insulins. BD MicroFine 5 mm: £9.69 per 100 needles resulting in a needle price of £0.097; Aviva test strips: £16.09 per 50 units^15^ and Fastclix lancets: £5.90 per 204 units^15^ resulting in a SMBG test cost of £0.35.

#### Alternate cost analyses

The cost data used in calculations performed included the costs of insulin pump set changes reported to be required by some carers when managing their child’s ketosis at home.^7^ As patients in the BEGIN YOUNG 1 study were exclusively on multiple daily injection therapy,^14^ additional sensitivity analyses were conducted to estimate mean annual costs per patient with insulin pump data removed.

## Results

### Main cost analysis

**Figure 2:**
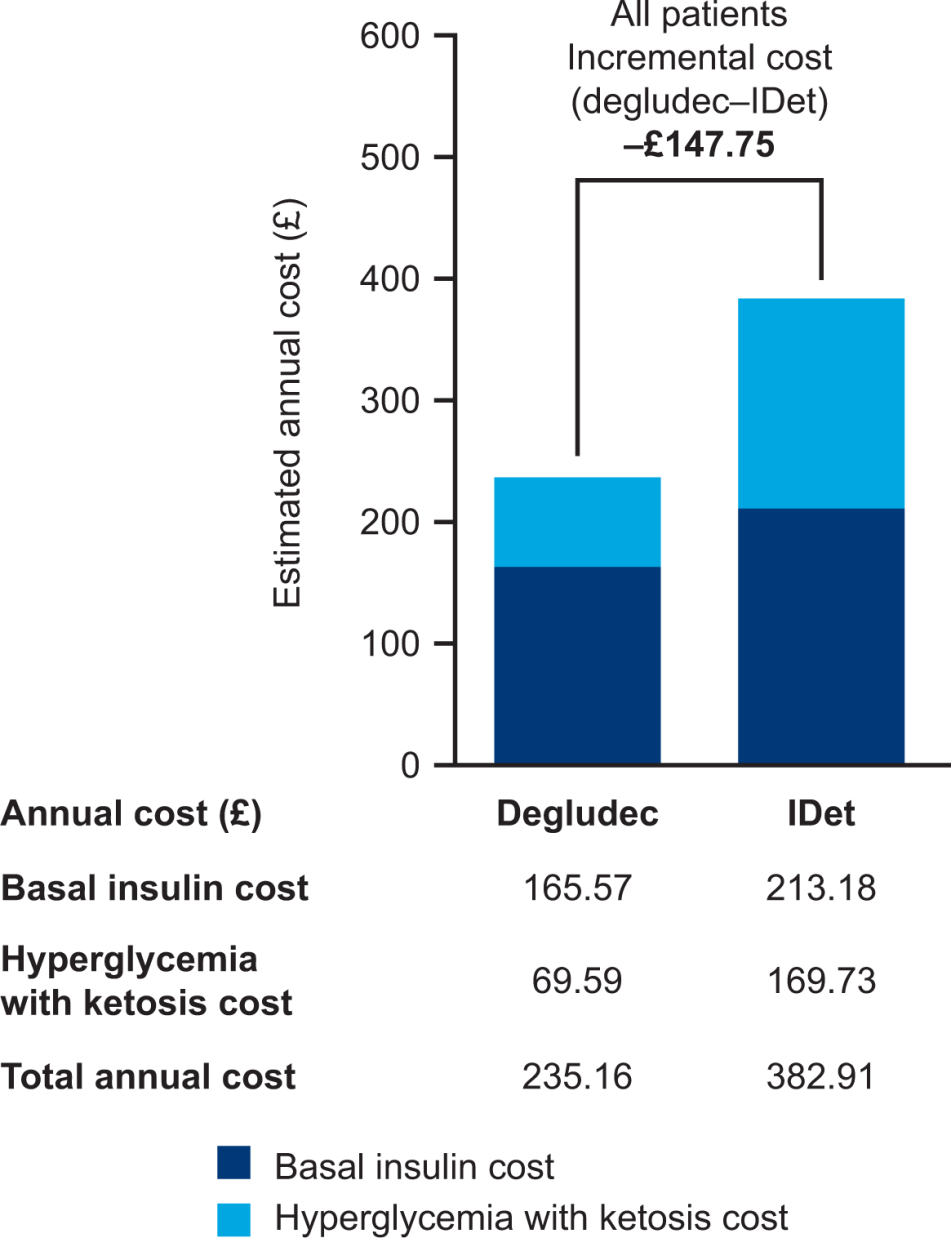
Main cost analysis of degludec compared with IDet in children and adolescents with type 1 diabetes. IDet, insulin detemir.

The mean annual cost per patient was estimated at £235.16 for degludec vs £382.91 for IDet (figure 2). This annual saving of £147.75 per patient in favor of degludec included a 22% (£47.61) reduction in basal insulin cost and a 59% (£100.14) reduction in the cost of hyperglycemia with ketosis events.

### Sensitivity analyses

#### Age group analyses

**Figure 3:**
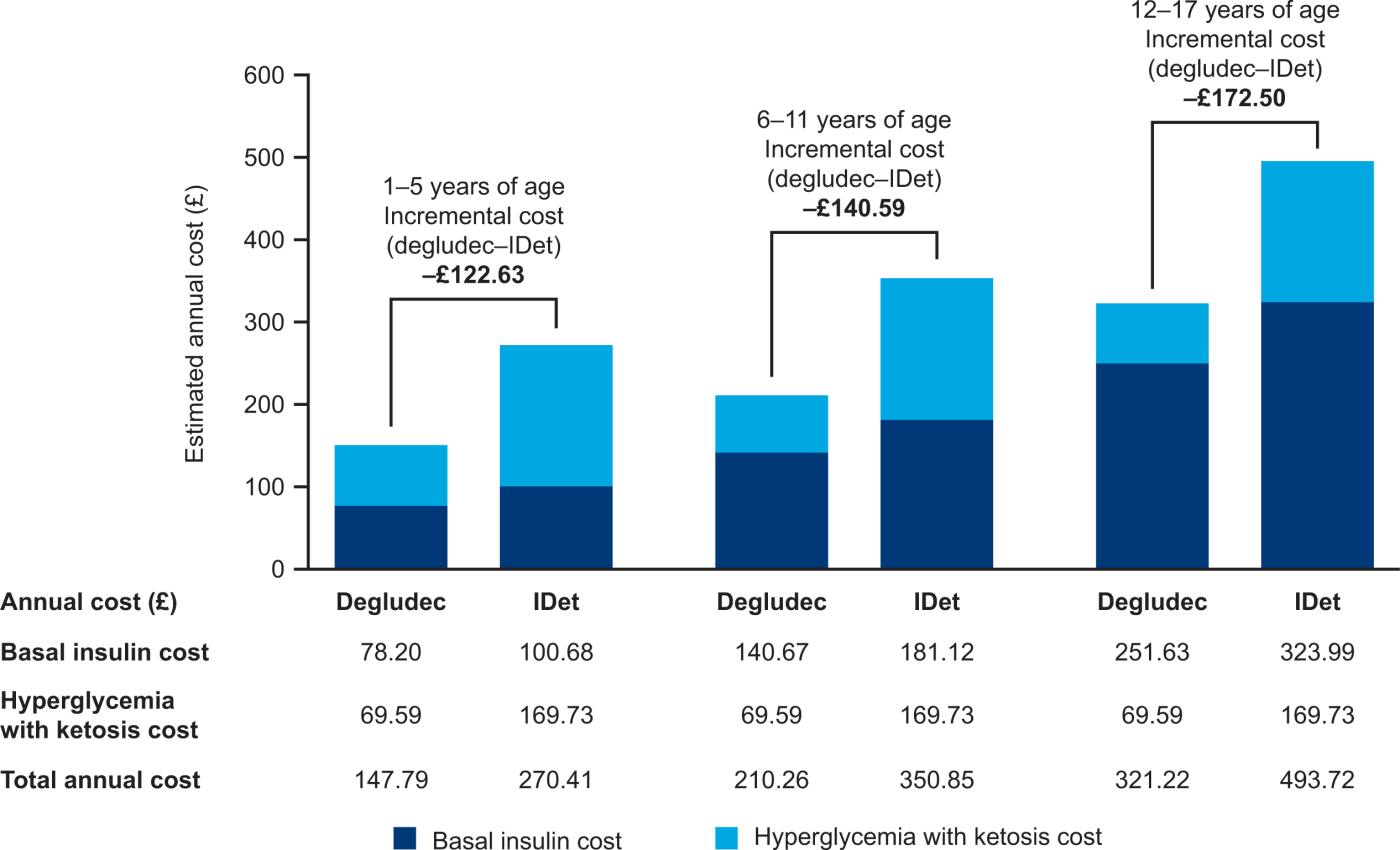
Age group analyses of degludec compared with IDet in children and adolescents with type 1 diabetes. IDet, insulin detemir.

In the age groups (1–5, 6–11 and 12–17 years of age), annual cost savings per patient were £122.63, £140.59 and £172.50, respectively, all in favor of degludec compared with IDet (figure 3).

#### Scenario analyses

**Figure 4:**
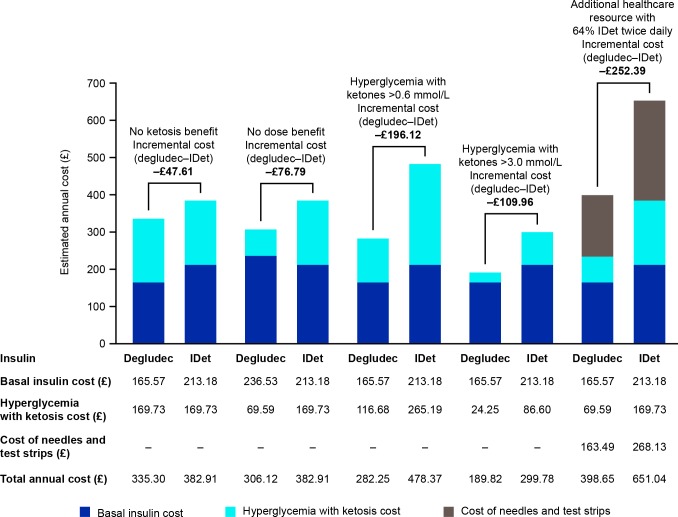
Scenario analyses of degludec compared with IDet in children and adolescents with type 1 diabetes. IDet, insulin detemir.

When no ketosis and no dose benefits were considered, an annual saving of £47.61 and £76.79 per patient in favor of degludec were estimated, respectively. When using ketone concentrations of >0.6 mmol/L (10.8 mg/dL) and >3.0 mmol/L (54 mg/dL) as the cut-offs for ketosis events, annual cost savings per patient of £196.12 and £109.96, respectively, were estimated in favor of degludec. When the costs of the additional healthcare resources required by 64% of patients administering IDet twice daily were considered, an annual saving of £252.39 per patient in favor of degludec was estimated (figure 4).

#### Alternate cost analyses

When the cost of insulin pump set changes was removed, costs calculated in the main analysis were slightly reduced (online supplementary figure 1). Compared with the main cost analysis, the estimated mean annual cost per patient for degludec reduced from £235.16 to £232.00, and that for IDet reduced from £382.91 to £375.21. This reflects an overall annual saving of £143.21 per patient in favor of degludec. Costs associated with the age group and scenario analyses were similarly reduced (online supplementary figures 2 and 3).

## Discussion

This cost analysis showed that degludec once daily provided an annual saving of £147.75 per patient, compared with IDet once daily/twice daily in children and adolescents with T1D in the UK; this annual saving with degludec once daily versus IDet once daily/twice daily was observed in the three age groups (1–5, 6–11 and 12–17 years of age). The scenario analyses demonstrated the robustness of the results with regard to parameter changes. These substantial cost savings were driven by the relative reductions in the frequency of hyperglycemia with ketosis and the basal insulin dose with degludec versus IDet.

Degludec is associated with low day-to-day variability in blood glucose-lowering profile,^13 17 18^ which can lead to a reduced rate of hypoglycemia, compared with other long-acting insulin analogs, at equivalent levels of glycemic control.^19–24^ Previous economic evaluations of degludec in adults with diabetes have taken into account its hypoglycemia benefit and shown that degludec was either cost-effective or dominant compared with insulin glargine 100 and 300 units/mL in adults with T1D or type 2 diabetes.^25 26^ However, no significant treatment difference in hypoglycemia risk was observed in BEGIN YOUNG 1, as children and adolescents were not titrated to the same level of glycemic control as adult patients in other randomized controlled trials.^14 19–23^ Due to this, rates of hypoglycemia were discounted from the present analysis.

The risk of DKA in children and adolescents with established T1D is up to 10% per patient-year.^27^ Depending on the severity of ketosis events and the experience of the pediatric carers, hyperglycemia with ketosis in children and adolescents with T1D may be managed at home and/or using healthcare facilities, leading to further financial burden on top of the prescription costs of insulin analogs.^7^ The cost savings achieved, in both basal insulin and ketosis events, along with clinical benefits of degludec versus IDet evidently facilitate healthcare providers’ ability to maximize health outcomes with restricted budgets.

Although indirect costs were not investigated in the current study, the lower rate of hyperglycemia with ketosis with degludec versus IDet in children and adolescents with T1D^14^ may provide further, indirect economic advantages through reduced resource use and improved work productivity of the pediatric carers.^7^ In addition, as it has been established that a numerically lower bolus insulin dose was required with degludec compared with IDet at the end of the BEGIN YOUNG 1 trial (0.55 vs 0.58 units/kg with IAsp),^14^ it is likely that this could contribute to further annual savings with degludec compared with IDet.

There are some limitations with the present study. First, a cost analysis was conducted instead of a cost-effectiveness analysis, where the health impact of complications with regard to quality-adjusted life-year (QALY) are quantified. While there is no well-established evidence linking reduced episodes of hyperglycemia with a QALY benefit, it is reasonable to assume that the reduced rates of hyperglycemia with ketosis with degludec versus IDet would result in a positive QALY gain. Therefore, it is expected that lower costs and higher QALYs could be achieved with degludec versus IDet (equivalent to a dominant incremental cost-effectiveness ratio). Evidence from different sources has informed this cost analysis and several estimates and assumptions made to perform calculations. Doses of degludec were calculated using dose ratios from the BEGIN YOUNG 1 trial.^14^ Data on numbers of events of hyperglycemia with ketosis were taken from two separate studies and events associated with degludec were estimated based on RRs. Events with ketone concentrations >1.5 mmol/L were estimated using data from BEGIN YOUNG 1.^14^ For the scenario sensitivity analyses, events with ketone concentrations >0.6 and >3.0 mmol/L were estimated from an analysis of two randomized trials^16^ and extrapolated to the BEGIN YOUNG 1 patient population. We have assumed that resources reported as required to treat hyperglycemia with ketosis in children at home and in NHS facilities, including a number of insulin pump set changes which may not be applicable, are generalizable to the BEGIN YOUNG 1 population.^7^ To remove the costs of pump set changes, however, could create bias, as costs associated with other components, such as test and ketone strips, could potentially be higher for non-pump users. The sensitivity analyses performed with the costs of insulin pump data removed reveals only a minor reduction in cost.

This study has several strengths. The clinical data from BEGIN YOUNG 1 were highly generalizable to the global population of pediatric patients with T1D using multiple daily injection therapy, due to the multinational nature and broad age range of this trial population.^14^ Results from the present study can be used to calculate the annual budget impact of treatment with degludec or IDet for a defined local patient population, whereby the annual cost per patient is simply multiplied by the number of patients treated. The findings from the present study could potentially be generalized to other countries with similar socioeconomic profiles and healthcare systems.

In conclusion, the lower dose requirement and risk of hyperglycemia with ketosis events associated with degludec drive appreciable annual cost savings compared with IDet in children and adolescents with T1D in a UK setting.
