# Supplementary material for: Cost analysis of insulin degludec in comparison with insulin detemir in treatment of children and adolescents with type 1 diabetes in the UK
Source: BMJ Open Diabetes Res Care. 2019 Sep 3;7(1):e000664. doi: 10.1136/bmjdrc-2019-000664 (PMC6731813; doi:10.1136/bmjdrc-2019-000664)
Supplement: Supplementary data [file bmjdrc-2019-000664supp001.pdf]

Supplementary material

Cost analysis of insulin degludec in comparison with insulin detemir in treatment of children and adolescents with type 1 diabetes in the UK

Nandu KS Thalange, Jens Gundgaard, Witesh Parekh, Deniz Tutkunkardas

Thalange N, *et al. BMJ Open Diab Res Care* 2019; 7:e000664. doi: 10.1136/bmjdrc-2019-000664

Supplementary Table 1: Unit costs for healthcare resources

Resource	Cost	Description	Reference
Blood ketone test	£1.50/strip	GlucoRx HCT Ketone testing strips (GlucoRx Ltd) 10 strips, £14.95/pack	MIMS [1]
Urine ketone test	£0.05/strip	GlucoRx KetoRx Sticks 2GK testing strips (GlucoRx Ltd). 50 strips, £2.25/pack	MIMS [1]
Blood glucose test	£0.05/strip	Medi-Test Glucose testing strips (BHR Pharmaceuticals Ltd). 50 strips, £2.33/pack	BNF [2]
Insulin	£0.01/unit	NovoRapid@ 100 units/mL solution for injection 10 mL vial (Novo Nordisk). 1 vial, £14.08	BNF [2]
Insulin pump set change	£12.06	Average cost of insulin infusion set	NICE TA151 [3]
Nurse-led phone service	£14.83	Specialist nurse unit cost per 10 minutes	PSSRU [4]
Nurse- or doctor-led phone service	£13.69	Weighted average of HCP unit cost per 10 minutes:<br>Specialist nurse (60%)*<br>Consultant (19%)*<br>Registrar (15%)*<br>Junior doctor (6%)*	PSSRU [4]
Non-HCP-led phone service	£1.63	Average emergency medical dispatcher salary, AfC band 2, assuming a 40-hour week with phone calls lasting 10 minutes*	National Careers Service [5]
Ambulance use	£98.71	Weighted average cost for ambulance codes ASC1, ASH1, ASS01, ASS02	Department of Health [6]
A&E admission	£148.36	Weighted average cost for all Emergency Medicine codes	Department of Health [6]
Hospital ward admission	£434.91	Weighted average cost for Non-Elective Short Stay hyperglycemia codes KB02G, KB02H, KB02J, KB02K	Department of Health [6-7]

Community healthcare services	£34.00	Weighted average of:<br>Specialist nurse unit cost per hour (50%)*<br>GP unit cost for 11.7 minute appointment (50%)*	PSSRU [4]
Follow-up face-to-face appointments	£24.38	Weighted average of HCP salaries over 15 minutes:<br>Specialist nurse unit cost per hour (50%)*<br>Consultant unit cost per hour (50%)*	PSSRU [4]

*Weighting based on time reported in HCP and patient/caregiver survey data or NHS workforce statistics

A&E, Accident & Emergency; AfC, Agenda for Change; BNF, British National Formulary; GP, general practitioner; HCP, healthcare professional; NHS, National Health Service; NICE TA, National Institute for Health and Care Excellence Technology Appraisal; PSSRU, Personal Social Services Research Unit

References

1. Monthly Index of Medical Specialties. Available from: https://www.mims.co.uk [accessed 18 June 2018]

2. British National Formulary. Available from: https://www.medicinescomplete.com [accessed 18 June 2018]

3. NICE. Continuous subcutaneous insulin infusion for the treatment of diabetes mellitus [TA151]. Available from: https://www.nice.org.uk/guidance/TA151 [accessed 18 June 2018]

4. Personal Social Services Research Unit. Unit costs of Health and Social Care 2017. Available from: https://www.pssru.ac.uk/project-pages/unit-costs/2017/index.php [accessed 18 June 2018]

5. National Careers Service. Emergency medical dispatcher. Available from: https://nationalcareersservice.direct.gov.uk/job-profiles/emergency-medical-dispatcher [accessed 18 June 2018]

6. Department of Health. NHS reference costs 2015 to 2016. Available from: https://www.gov.uk/government/publications/nhs-reference-costs-2015-to-2016 [accessed 18 June 2018]

7. Department of Health. NHS reference costs 2015 to 2016. Available from: https://improvement.nhs.uk/resources/reference-costs/ [accessed 18 June 2018]

Thalange N, *et al. BMJ Open Diab Res Care* 2019; 7:e000664. doi: 10.1136/bmjdrc-2019-000664

Supplementary Figure 1: Main cost analysis of degludec compared with IDet in children and adolescents with T1D (costs of changing insulin pump sets removed)

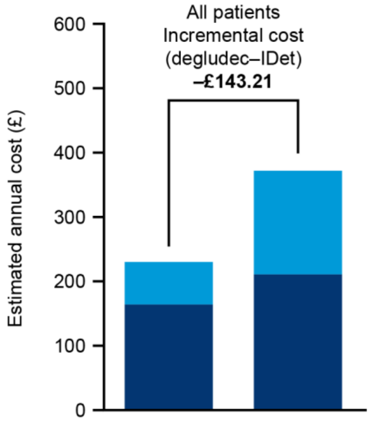

Annual cost (£)	Degludec	IDet
Basal insulin cost	165.57	213.18
Hyperglycemia with ketosis cost	66.43	162.03
Total annual cost	232.00	375.21

Basal insulin cost
Hyperglycemia with ketosis cost

IDet, insulin detemir; T1D, type 1 diabetes.

Supplementary Figure 2: Age group analyses of degludec compared with IDet in children and adolescents with T1D (costs of changing insulin pump sets removed)

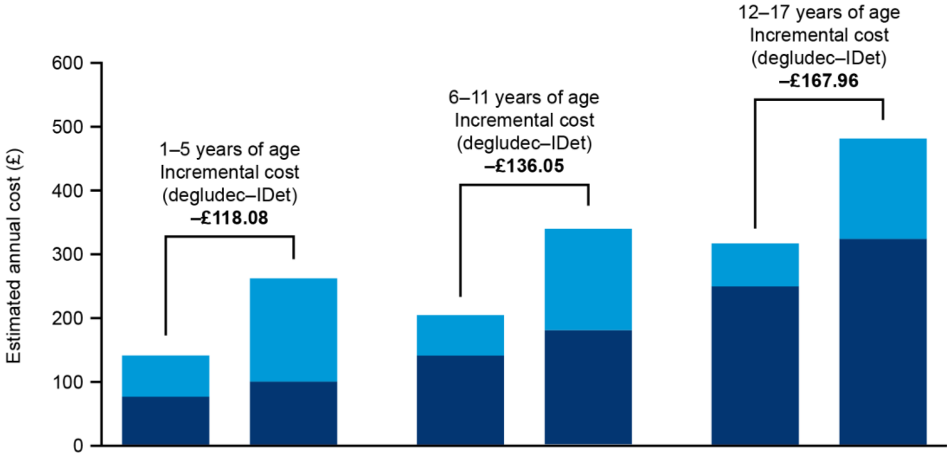

Annual cost (£)	Degludec	IDet	Degludec	IDet	Degludec	IDet
Basal insulin cost	78.20	100.68	140.67	181.12	251.63	323.99
Hyperglycemia with ketosis cost	66.43	162.03	66.43	162.03	66.43	162.03
Total annual cost	144.63	262.71	207.10	343.15	318.07	486.02

■ Basal insulin cost ■ Hyperglycemia with ketosis cost

IDet, insulin detemir; T1D, type 1 diabetes.

Thalange N, *et al. BMJ Open Diab Res Care* 2019; 7:e000664. doi: 10.1136/bmjdrc-2019-000664

Supplementary Figure 3: Scenario analyses of degludec compared with IDet in children and adolescents with T1D (costs of changing insulin pump sets removed)

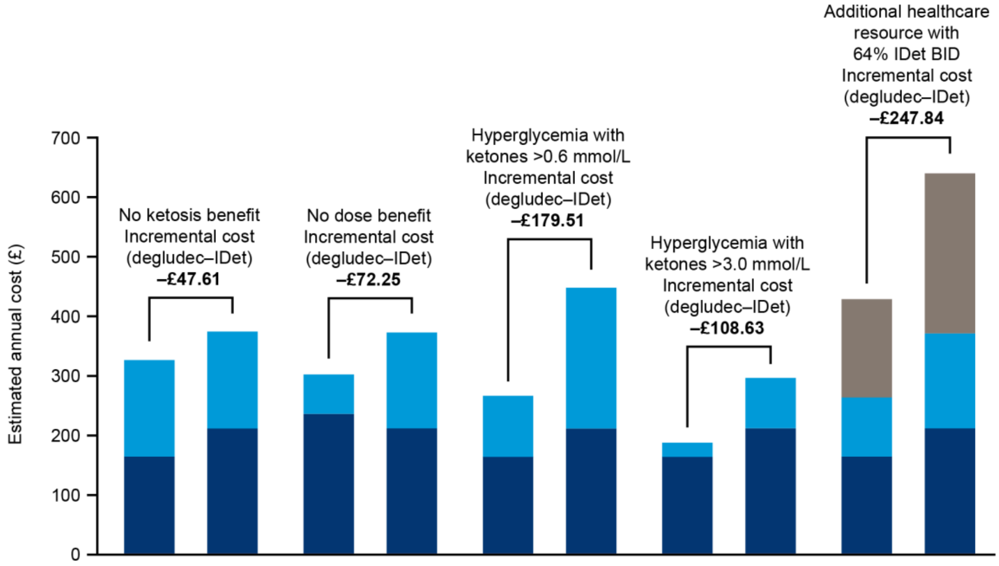

Insulin	Degludec	IDet	Degludec	IDet	Degludec	IDet	Degludec	IDet	Degludec	IDet
Basal insulin cost (£)	165.57	213.18	236.53	213.18	165.57	213.18	165.57	213.18	165.57	213.18
Hyperglycemia with ketosis cost (£)	162.03	162.03	66.43	162.03	103.63	235.53	23.73	84.75	66.43	162.03
Cost of needles and test strips (£)	–	–	–	–	–	–	–	–	163.49	268.13
Total annual cost (£)	327.60	375.21	302.96	375.21	269.20	448.71	189.30	297.93	395.50	643.34

■ Basal insulin cost ■ Hyperglycemia with ketosis cost ■ Cost of needles and test strips

BID, twice daily; IDet, insulin detemir; T1D, type 1 diabetes.
